# The Causes of Acute Fever Requiring Hospitalization in Geriatric Patients: Comparison of Infectious and Noninfectious Etiology

**DOI:** 10.4061/2010/380892

**Published:** 2010-08-12

**Authors:** A. Atahan Cagatay, Fatih Tufan, Fehmi Hindilerden, Sibel Aydin, Omer Celal Elcioglu, Asli Karadeniz, Nilufer Alpay, Suut Gokturk, Ozer Taranoglu

**Affiliations:** ^1^Istanbul University, Istanbul Faculty of Medicine, Department of Infectious Diseases and Clinical Microbiology, PB 34390, Fatih, Istanbul, Turkey; ^2^Istanbul University, Istanbul Faculty of Medicine, Department of Internal Medicine, PB 34390, Fatih, Istanbul, Turkey

## Abstract

*Introduction*. Infectious diseases may present with atypical presentations in the geriatric patients. While fever is an important finding of infections, it may also be a sign of noninfectious etiology. *Methods*. Geriatric patients who were hospitalized for acute fever in our infectious diseases unit were included. Acute fever was defined as presentation within the first week of fever above 37.3°C. *Results*. 185 patients were included (82 males and 103 females). Mean age was 69.7 ± 7.5 years. The cause of fever was an infectious disease in 135 and noninfectious disease in 32 and unknown in 18 of the patients. The most common infectious etiologies were respiratory tract infections (*n* = 46), urinary tract infections (*n* = 26), and skin and soft tissue infections (*n* = 23). Noninfectious causes of fever were rheumatic diseases (*n* = 8), solid tumors (*n* = 7), hematological diseases (*n* = 10), and vasculitis (*n* = 7). A noninfectious cause of fever was present in one patient with no underlying diseases and in 31 of 130 patients with underlying diseases. *Conclusion*. Geriatric patients with no underlying diseases generally had infectious causes of fever while noninfectious causes were responsible from fever in an important proportion of patients with underlying diseases.

## 1. Introduction

The geriatric population, defined as people over the age of 65, comprised 6.2% of the world population in 1992 and is estimated to reach 20% by 2050 [1]. Aging is associated with numerous chronic illnesses and comorbid conditions [2, 3], polypharmacy and immunosuppressive medications, and changes in the immune system [2, 4]. Aging increases the susceptibility to infection. Moreover, both morbidity and mortality for many infections may be several-fold higher in the elderly with respect to the young. The susceptibility to infection is influenced by numerous elements. Although prevention is the most effective measure to reduce morbidity, mortality, and the expense of infections in the elderly, the prompt diagnosis and initiation of appropriate supportive and antimicrobial therapy is also a critical strategy for the management of infection in the geriatric patient. 

Infection often presents itself in atypical forms in the geriatric population. Fever, being a cardinal symptom in the manifestation of infections, is an important clue for the diagnosis of noninfectious diseases such as rheumatic disease and malignancy as well. Although many studies have thoroughly investigated the causes of fever of unknown origin, there are no studies up to date about the etiologies of acute fever in the geriatric population.

## 2. Methods

Geriatric patients hospitalized at the Clinical Microbiology and Infectious Disease department of Istanbul Faculty of Medicine between 1999 and 2007 with the complaints of high fever and waning general health status were enrolled in this study. Advanced age and very advanced age were defined as 65 and above, 85 and above, respectively [5, 6]. Patients with upper respiratory tract infection were excluded. Patients referring to the clinic within the first seven days of fever which was measured to be above 37.3°C were accepted to have acute fever [7]. In this group of geriatric patients evaluated for acute fever, our study aimed to determine the underlying diseases, the most useful clinical and laboratory findings for the accurate diagnosis. In addition, the mortality rates for specific infections and the risk factors contributing to high mortality rates were ascertained.

## 3. Results

185 patients (82 male, 103 female) were eligible for the study. The median age was 69.7 ± 7.5 (65–90). The cause of the fever was infectious disease in 135 patients (72.9%), noninfectious disease in 32 patients (17.2%); 18 (9.7%) patients were undiagnosed. Of the 135 patients with infection, 46 (24.8%) had lower respiratory tract infection (41 community acquired pneumonia, 5 pulmonary tuberculosis), 26 (14%) had urinary tract infection (UTI), 23 (12.4%) had skin and soft tissue infection (10 cellulitis, 4 erysipelas, 2 diabetic foot infection, 3 pyomyositis, 1 carbuncle, 1 ophthalmic zoster, 2 thrombophlebitis), 3 (1.6%) had orthopedic infection (2 prosthetic infection, 1 septic arthritis), 7 (3.7%) had intra-abdominal infection (3 intraabdominal abscess, 2 liver abscess, 1 cholecystitis, 1 cholangitis), 10 (5.4%) had central nervous system infection, 1 (0.5%) had acute osteomyelitis, 5 (2.7%) had cardiovascular system infection, 6 (3.2%) had brucellosis, 3 (1.6%) had disseminated tuberculosis, and 1 (0.5%) had rickettsial disease. Of the 32 patients with noninfectious disease, 8 (4.3%) had rheumatologic disease (5 Adult's Still disease, 2 FMF, 1 seronegative arthritis), 7 (3.7%) had solid tumor (3 bronchogenic carcinoma, 3 colon cancer, 1 rectum cancer), 10 (%3.7) had hematological disease (5 NHL, 4 CLL, 1 AML), 7 (3.7%) had vasculitis (7 temporal arteritis).

The patients' history revealed the following underlying diseases in our study group; 45 (24.3%) had diabetes mellitus, 35 (18.9%) had hypertensive ischemic heart disease, 28 (15.1%) had hematological disease (8 CLL, 7 NHL, 7 AML, 4 CML, 1 Multiple myeloma, 1 MDS), 28 (15.1%) had history of surgical intervention within the past 6 months, 22 (%11.9) had solid tumor (6 lung, 4 bladder, 2 colon, 2 rectum, 2 prostate, 4 brain -2 malign brain tumor, 1 pituitary adenoma, 1 meningioma, 1 mesothelioma, 1 nasopharnyx), 22 (11.9%) had chronic renal insufficiency, 13 (7%) had chronic pulmonary infection, and 4 (2.1%) had chronic liver disease. With respect to nondiabetic patients, the diabetics had significantly longer mean period of hospitalization (21.56 ± 16.52 versus 16.73 ± 12.81 days *P* = .04), significantly higher neutrophil counts (9765.9 ± 1266.7/ml versus 7197.1 ± 6266.7/ml *P* = .003), and significantly lower levels of albumin (2.9 ± 0.7 gr/dl versus 3.1 ± 0.4 gr/dl *P* = .03). Among the diabetics, the cause of the fever was infectious diseases in 37 patients (82.2%), noninfectious disease in 6 patients (13.3%). The source of fever could not be identified in 2 patients (4.4%). 

The mean age of patients with infectious disease and noninfectious disease as the source of fever was 70.1 ± 7.6 and 68.5 ± 7 respectively. The mean period of hospitalization was 17.78 ± 13.51 days, and 18.34 ± 15.19 days respectively.

51 patients had no history of predisposing factors except for advanced age. At least one underlying disease (diabetes mellitus, hypertensive ischemic heart disease, solid tumor, hematological malignity, chronic renal failure, previous surgical intervention, chronic obstructive pulmonary disorder, chronic liver disease, corticosteroid administration over two weeks) was present in the remaining patients. In advanced aged patients with no history of underlying disease (*n* = 51), infection was identified as the cause of fever in 36 (70%) while in 1 (2%) patient noninfectious etiology was documented. On the other hand, in advanced aged patients with history of at least one underlying disorder (*n* = 134), the cause of fever was infection in 99 (73.8%) and noninfectious disease in 31 (23.1%) (Table 1).

The frequency of signs and symptoms accompanying fever was also documented. The most common findings were rales (*n* = 83), tachypnea (*n* = 67), pallor (*n* = 57), tachycardia (*n* = 56), cough (*n* = 46), and hepatomegaly (*n* = 40). Dyspnea, rales, and confusion were significantly more frequent in patients with infection identified as the cause of fever (*P* = .02, *P* = .04, *P* = .01, resp.) (Table 2).

Laboratory analysis revealed significantly higher mean neutrophil count, total iron binding capacity, and MCV for infectious etiologies (*P* < .03, *P* < .04 and *P* < .03, resp.) while erythrocyte sedimentation rate and alkaline phosphatase were significantly higher for noninfectious etiologies (*P* = .03, *P* = .04, resp.) (Table 3). In addition, erythrocyte sedimentation rate (ESR) over 100 mm/hour was significant for noninfectious etiologies of fever (*P* < .001). 

13 (7.02%) patients in the study group died. The cause of death was infectious disease in 11 patients (9 community acquired pneumonia, 1 bacterial meningitis, 1 skin and soft tissue infection) while the cause was noninfectious disease in 2 patients (2 hematological malignancies). The most common underlying diseases were not more common in the deceased group (diabetes mellitus 4/13 versus 41/172; hypertensive ischemic heart disease 3/13 versus 32/172, and hematological diseases 4/13 versus 24/172, *P* > .05 for all comparisons). Pneumonia was present in 10 (20%) geriatric patients with no underlying diseases and 31 (23%) patients with underlying diseases. In this study population, 9 of 41 patients with pneumonia died (22% mortality rate). The mean age of the deceased and survived patients was similar (70.6 ± 5.8 versus 69.6 ± 7.6, *P* > .05).

## 4. Discussion

### 4.1. Aging and Infectious Diseases

The mean age for populations is currently rising. By 2030, every one of five individuals in the United States will be over age 65. The most rapidly growing segment of this population is those over age 85 years [5, 8]. In the United States, the average life expectancy at 65 is 18.9 years, 11 years at 75, and 7 years at 85 [9]. The waning function of cardiovascular, respiratory, and renal systems in the elderly is by itself a frequent cause of medical reference. It is important to acknowledge that the malfunction of the mentioned systems causes an immunocompromised state. This prepares the grounds for many infectious and noninfectious diseases contributing to the increased morbidity and mortality in this group of patients. 

Aging causes the increased susceptibility to infection [10–13]. The decline is prominent in cellular immunity. Because of continuous antigenic stimulation circulating memory T cells increase while naïve T cells decrease due to age-associated involution of thymus [12, 13]. Response to foreign antigens is also diminished because of increasing lack of regulatory control of T cells on B cells [13]. On the other hand, neutrophils, macrophages, and neutral killer cells continue to function reasonably well [13]. Malnutrition, poor circulatory, and breakdown of natural mechanical barriers which are commonly seen in the elderly also contribute to infections in this population [13]. Moreover, both morbidity and mortality for many infections may be several-fold higher in the elderly with respect to the young [14]. Physiological reserves are diminished in the geriatric population. This condition puts the patient at risk for certain disease states, examples of which include decreased cough reflex leading to aspiration pneumonia, impaired arterial and venous circulation, and compromised wound healing, making cellulitis a common infection [1]. Geriatric patients are susceptible to infections in a similar fashion to that of younger individuals; however, there are a few exceptions. First, the immune systems of elderly patients may not respond as readily as those of their younger counterparts, because of either the well-known diminution of activity or the suppression due to intercurrent infection or other treatment. Secondly, elderly patients may live in a different climate, preferring warmer temperatures and increased humidity. Third, their behavior may change, making them susceptible to new infections [15].

### 4.2. Infectious Causes of Fever

This study identified lower respiratory tract, urinary tract, and skin and soft tissue infections as the most common infectious causes of fever in the geriatric group. Although pneumonia in the elderly patient is not reported as the most common cause of acute fever or fever of unknown origin, it is an important cause of morbidity and mortality in this group [16]. Diseases of the respiratory tract have long been associated with morbidity and mortality among the elderly population. Although pneumonia accounts for 13% to 48% of infections among nursing home residents, mortality rates of those admitted to the hospital are as high as 44% [17]. It is also the second most common cause of bacteremia in nursing homes. This study discriminated lower respiratory tract infections as one of the most common diseases and a very frequent cause of infection in the geriatric patients. In addition, our observations showed that pulmonary tuberculosis was not the cause of fever in the geriatric patient with no underlying illness. 

UTI remains as one of the leading causes of infection in elderly patients. Among otherwise healthy geriatric patients, rates for UTI range from 5% to 30%, with higher rates seen in advanced age group. Among institutionalized patients, the prevalence rates increase remarkably. 17% to 55% of women and 15% and 31% of men are reported to be bacteriuric in one study [18]. The frequency of UTI as the cause of fever in this study was 14%; UTI was relatively more frequent in those with underlying disease.

In this study group, 14% of patients had skin and soft tissue infection identified as source of fever. It has been estimated that the prevalence of skin and soft tissue infection in the long-term care facility is 5% [1] Cellulitis is both more common and severe in the geriatric patient with respect to the young. Cellulitis in the elderly is often attributed to chronic venous insufficiency, peripheral vascular disease, malnutrition, and trauma [19].

### 4.3. Noninfectious Causes of Fever

Rheumatologic diseases are the most common noninfectious causes of acute fever [20]. Furthermore, patient's age plays an important role in many different forms of rheumatic disease [16]. Vasculitis, the most common rheumatologic cause of fever in this study, is associated with inflammation of vessels. Vasculitis often presents with fever or/and with fever of unknown origin [21–23]. The most common form of systemic vasculitis seen in humans, giant cell arteritis (GCA), occurs almost exclusively in people over the age of 50. For this and many other forms of vasculitic disease that can develop in older patients, the challenges of diagnosis and treatment can be further compounded by the presence of comorbid diseases and concomitant medications. Temporal arteritis, a form of giant cell arteritis, was the most common vasculitic disease identified as the cause of fever in this study group. Adult's Still disease was the second most frequent rheumatologic cause of fever in this study. In this study, it was observed that noninfectious etiologies were more frequently identified as cause of fever in patients with known underlying disease. This finding may be attributed to the relatively more severe immunocompromised state in these patients.

### 4.4. Clinical Presentation

The analysis of the symptoms accompanying fever showed that dyspnea, presence of rales, and mental confusion were significantly more common in infectious etiologies (Table 2). This could be explained by the increased frequency of infectious disease in the elderly with underlying disease and the general waning health status due to presence of fever. Laboratory analysis showed that mean neutrophil counts were significantly higher in infections while mean ESR and ALP levels were significantly more elevated in noninfectious diseases. Presence of ESR over 100 mm/hour was significantly associated with noninfectious disease. The high levels of acute phase reactants accompanying infection are expected findings. The higher levels of ALP in noninfectious disease were attributed to the infiltrative behavior of diseases such as vasculitis and malignancy.

### 4.5. Association with Background Diseases

The most common underlying disease in our study group was diabetes mellitus followed by hypertensive ischemic heart disease and hematological disease. Cardiovascular, respiratory, and renal insufficiencies are more readily encountered in the geriatric group [5]. An important data gained from this study was the presence of malignity as the underlying disease in 11.9% of patients requiring hospitalization for acute fever. This study showed that infectious disease was the cause of 82.2% of diabetic patients evaluated for acute fever. This finding is not surprising when the decreased immune defense associated with diabetes is regarded. Adherence, chemotaxis, and intracellular killing of neutrophils, monocytes, and lymphocytes; reduced cell-mediated immune responses; abnormal delayed type hypersensitivity responses are known factors for described abnormalities in immune functions associated with diabetes mellitus [24]. However, humoral immunity is relatively intact in diabetic patients [24]. Patients with diabetes commonly develop oral candidiasis, urinary tract infections, skin and soft tissue infections, osteomyelitis, tuberculosis, pneumonia related to gram negative bacteria, cholecystitis, and gastrointestinal infections [24].

Noninfectious causes of fever were more commonly identified (23.1%) in patients with known underlying disease with respect to those with no underlying risk factors except for old age.

### 4.6. Prognosis

Thirteen patients in this study died. The cause of death was infection in most cases (84.6%). Mortality was not associated with age or presence of underlying diseases in this study population. The reason for this may be the low mortality rate in these patients. Infectious diseases are reported to be the cause of acute fever requiring hospitalization in 75% of patients. These patients are likely to have high morbidity and mortality rates. It is important to remember that infectious diseases especially with the copresence of known underlying diseases may show rapid progression. Pneumonia may be an important cause of mortality in the elderly patients.

## 5. Conclusion

As the life expectancy increases, the numbers of geriatric patients and their mean age are going to increase. In those patients with no underlying predisposing conditions except for age, an infectious etiology is to be sought. The evaluation must be prompt, and the treatment should begin as early as possible. In the elderly patient with underlying disease, it will be more rational to consider noninfectious etiologies as the cause of fever.

## Figures and Tables

**Table 1:** The etiologies of acute fever in geriatric patients with underlying disease and with no underlying disease.

	Geriatric patients (*n* = 51)	Geriatric patient with underlying disease (*n* = 134)
*Infectious diseases *(*n* = 135,72.9%)	36 (70.5%)	99 (73.8%)
Lower respiratory tract infection (*n* = 46, 24.8%)	10 (20%)	36 (27%)
Pneumonia	10 (20%)	31 (23%)
Pulmonary tuberculosis		5 (4%)
Infection of skin and soft tissue (*n* = 26, 14%)	7 (14%)	19 (14%)
Orthopedic Infections (*n* = 3, 1.6%)	0	3 (2%)
Urinary tract infections (*n* = 26, 14%)	5 (10%)	21 (16%)
Central nervous system infection (*n* = 10, 5.4%)	4 (8%)	6 (4.5%)
Intrabdominal infections (*n* = 7, 3.7%)	2 (4%)	5 (4%)
Vascular system infections (*n* = 6, 3. 2%)	0	6 (4.5%)
Disseminated tuberculosis (*n* = 3, 1.6%)	0	3 (2%)
Brusellosis (*n* = 6, 3.2%)	6 (12%)	0
Ricketsial disease (*n* = 1, 0.5%)	1 (2%)	0
Acute osteomyelitis (*n* = 1, 0.5%)	1 (2%)	0

*Noninfectious diseases *(*n* = 32, 17.3%)	*1 *(2%)	31 (23.1%)
Rheumatologic diseases (*n* = 8, 4.3%)	1 (2%)	7 (5%)
Vasculitis (*n* = 7, 3.7%)	0	7 (5%)
Solid tumor (*n* = 7, 3.7%)	0	7 (5%)
Hematologic malignities (*n* = 10, 5.4%)	0	10 (7.5%)

*Undiagnosed diseases *(*n* = 14, 9.7%)	14 (27.4%)	4 (2.9%)

**Table 2:** Demographic data and frequencies of signs and symptoms accompanying acute fever.

	Infectious diseases (*n* = 135)	Noninfectious diseases (*n* = 32)	*P* value*
Mean age	70.1 ± 7.6	68.5 ± 7	.2
Mean duration of hospitalisation	2.54 ± 1.93	2.62 ± 2.17	.8
Cough (*n* = 46)	37	9	.2
Sputum (*n* = 28)	24	4	.1
Dyspnea (*n* = 27)	26	1	**.02**
Chest pain (*n* = 9)	5	4	.2
Palpitation (*n* = 3)	2	1	1
Abdominal pain (*n* = 22)	14	8	.3
Diarrhea (*n* = 7)	4	3	.4
Constipation (*n* = 4)	1	3	.06
Lymphadenomegaly (*n* = 29)	20	9	.4
Cervical	8	3	.8
Axillary	3	2	.6
Inguinal	2	0	.5
Intra-abdominal	2	1	1
Mediastinal	5	3	.4
Hepatomegaly (*n* = 40)	26	14	.2
Splenomegaly (*n* = 24)	15	9	.2
Jaundice (*n* = 3)	3	0	.5
Pallor (*n* = 57)	39	19	.3
Tachypnea (*n* = 67)	52	15	.3
Tachycardia (*n* = 56)	44	12	.4
Rales (*n* = 83)	67	16	**.04**
Ronchus (*n* = 26)	23	3	.06
Confusion (*n* = 27)	25	2	**.01**
Maculopapular rash (*n* = 11)	9	2	.7
Erythema (*n* = 29)	25	4	.1
Swelling (*n* = 26)	20	6	.8
Bruit (*n* = 32)	24	8	1
Arthritis (*n* = 7)	4	3	.3
Dysuria (*n* = 18)	16	2	.1
Costovertebral tenderness (*n* = 20)	17	3	.2

**Table 3:** Laboratory features in diseases causing acute fever in geriatric patients.

	Infectious diseases (*n* = 135)	Noninfectious diseases (*n* = 32)	*P* value
Leukocyte count	20184.4 ± 50.393	9093.6 ± 6194.5	.1
Neutrophil count	8615.2 ± 7648.6	5868 ± 3850.4	*.02*
Lymphocyte count	11132.2 ± 49635.2	1875.6 ± 4592.1	.2
Hemoglobin	10876 ± 2	10518 ± 2.7	.3
MCV	87.1 ± 7.5	82.1 ± 7.1	*.03*
Platelet count	266992.5 ± 237329.2	250229.9 ± 158682.7	.6
C-Reactive protein	124.7 ± 114.384	123.2 ± 109.5	.9
Erythrocyte sedimentation rate	66.6 ± 28.3	77.8 ± 33.7	**.03**
Ferritin	1006.4 ± 2229.1	763.1 ± 609.6	.7
Iron	46 ± 32	36.3 ± 17.7	.7
TDBK	245.7 ± 62.9	202.9 ± 47.4	**.04**
AST	43.9 ± 53.5	35 ± 25.1	.2
ALT	47.8 ± 72.9	38 ± 47.8	.3
ALP	209.4 ± 186	287 ± 253.8	**.04**
GGT	81.7 ± 112	89.4 ± 95.9	.7
Glucose	122.1 ± 49.1	113.2 ± 42.7	.2
Creatinine	1.1 ± 0.7	1.1 ± 0.8	.9
LDH	422.5 ± 725.2	427 ± 200.6	.9
Albumin	3.09 ± 0.5	3.1 ± 0.5	.3
Gama globulin	1.3 ± 0.5	1.4 ± 0.6	.3
Calcium	8.5 ± 0.7	8.6 ± 0.8	.5
Corrected calcium	9.3 ± 0.7	9.3 ± 0.7	.7
